# Reducing the Risk of Acrylamide and Other Processing Contaminant Formation in Wheat Products

**DOI:** 10.3390/foods12173264

**Published:** 2023-08-30

**Authors:** Navneet Kaur, Nigel G. Halford

**Affiliations:** Rothamsted Research, Harpenden AL5 2JQ, Hertfordshire, UK; navneet.kaur@rothamsted.ac.uk

**Keywords:** acrylamide, asparagine, food safety, free amino acids, hydroxymethylfurfuryl, Maillard reaction, polycyclic aromatic hydrocarbons, processing contaminants, reducing sugars, wheat

## Abstract

Wheat is a staple crop, consumed worldwide as a major source of starch and protein. Global intake of wheat has increased in recent years, and overall, wheat is considered to be a healthy food, particularly when products are made from whole grains. However, wheat is almost invariably processed before it is consumed, usually via baking and/or toasting, and this can lead to the formation of toxic processing contaminants, including acrylamide, 5-hydroxymethylfurfural (HMF) and polycyclic aromatic hydrocarbons (PAHs). Acrylamide is principally formed from free (soluble, non-protein) asparagine and reducing sugars (glucose, fructose and maltose) within the Maillard reaction and is classified as a Group 2A carcinogen (probably carcinogenic to humans). It also has neurotoxic and developmental effects at high doses. HMF is also generated within the Maillard reaction but can also be formed via the dehydration of fructose or caramelisation. It is frequently found in bread, biscuits, cookies, and cakes. Its molecular structure points to genotoxicity and carcinogenic risks. PAHs are a large class of chemical compounds, many of which are genotoxic, mutagenic, teratogenic and carcinogenic. They are mostly formed during frying, baking and grilling due to incomplete combustion of organic matter. Production of these processing contaminants can be reduced with changes in recipe and processing parameters, along with effective quality control measures. However, in the case of acrylamide and HMF, their formation is also highly dependent on the concentrations of precursors in the grain. Here, we review the synthesis of these contaminants, factors impacting their production and the mitigation measures that can be taken to reduce their formation in wheat products, focusing on the role of genetics and agronomy. We also review the risk management measures adopted by food safety authorities around the world.

## 1. Introduction

Wheat is a staple crop, consumed worldwide as a major source of starch and protein, accounting for 20% of the total calories consumed by humans. Hexaploid wheat, *Triticum aestivum*, generally known as “common” or “bread” wheat, is the most widely grown species worldwide and is used to make a variety of products, including bread, biscuits, breakfast cereals, flatbreads, cakes, pies, batter and savoury snacks. The tetraploid species, *T. turgidum* var. *durum*, contributes 6–7% of total wheat production and is mainly used for making pasta (it is often referred to as “pasta” as well as “Durum” wheat). It is cultivated mainly in hot, dry conditions. Some other species, such as tetraploid emmer wheat (*Triticum dicoccoides*, also known as *Triticum turgidum* subsp. *dicoccoides*), hexaploid spelt wheat (*Triticum spelta*) and diploid einkorn wheat (*Triticum monococcum*) are cultivated in relatively small, localised regions [1].

The production of food products from wheat grain requires processing, the first stage of which is the milling of the grain to produce flour. The principal components of the grain are the bran, embryo and endosperm, and these can be separated out in different milling fractions. About 70% of the flour derives from the endosperm, and in refined, white flour, the other components are removed. Wheat varieties are grouped according to their suitability for different end uses. In the UK, for example, a classification defined by UK Flour Millers (UKFM) is used [2], with the following groupings: Group 1, varieties suitable for bread-making, with consistent milling and baking performance; Group 2, varieties with bread-making potential but not suited to all grists; Group 3, soft varieties used for biscuits, breakfast cereals, cakes and similar products; Group 4, sub-grouped into hard and soft types and used mainly for animal feed and bioethanol, although incorporated into some grists for food use. The grain of Groups 1 and 2 varieties has 11–15% protein content, while the grain of soft varieties has 10% protein content or less.

**Figure 1:**
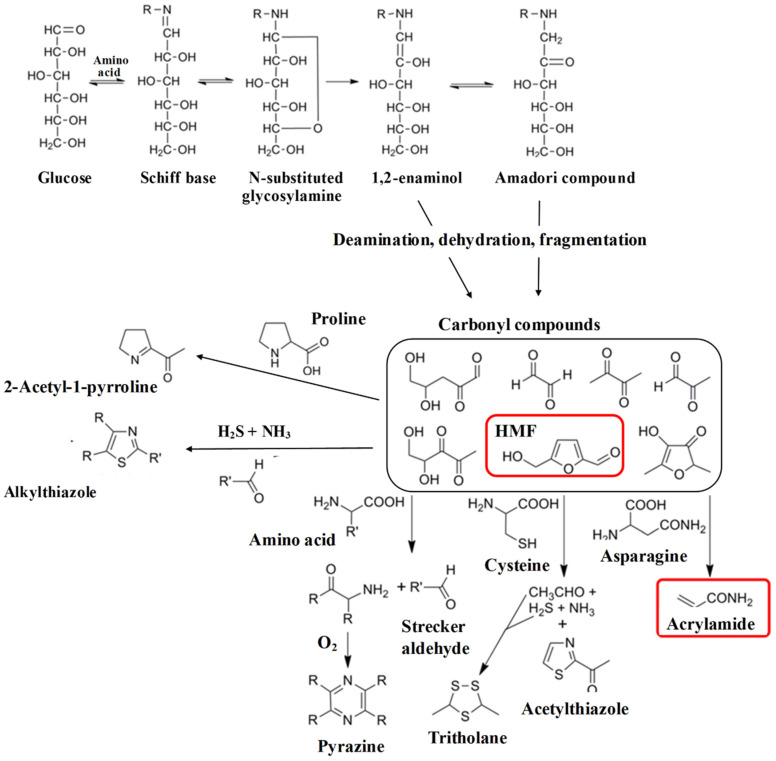
Simplified representation of the Maillard reaction showing the formation of acrylamide, HMF and some of the many other reaction products, many of which contribute to the flavour and aroma of fried, baked, roasted and toasted foods. The pathway shown involves the formation of an Amadori compound from a Schiff base produced by a reaction between glucose and an amino acid. A related Heyns compound is formed from fructose. The deamination, dehydration and fragmentation of these compounds give rise to highly reactive carbonyl compounds, including HMF. These may react with free amino acids again, giving rise to a plethora of products. Acrylamide and HMF are highlighted by red frames. Adapted from [5].

The mixing of flour with water and other ingredients, followed by baking and/or toasting, imparts texture, colour, flavour and aroma. In short, it turns the flour into a palatable food. Many of the compounds that are responsible for the colours, flavours and aromas associated with baked and toasted foods are produced in the Maillard reaction, a complex series of reactions involving free amino acids and reducing sugars. A simple representation of the Maillard reaction is given in Figure 1. However, high-temperature cooking and processing also lead to the production of undesirable processing contaminants via thermally-induced chemical reactions between constituents, as well as dehydration, caramelisation, lipid oxidation and other mechanisms. We define processing contaminants as substances that are produced in a food when it is cooked or processed, are not present or are present at much lower concentrations in the raw, unprocessed food, and are undesirable either because they have adverse effects on product quality or because they are potentially harmful [3]. The major processing contaminants of wheat products are acrylamide, furans and furanic compounds, of which 5-hydroxymethylfurfural (HMF) has received most attention to date, and polycyclic aromatic hydrocarbons (PAHs) [4].

The formation of these contaminants depends upon many factors, from the effects of variety selection and crop management on flour composition through to product recipes, measures taken by processors and food preparers, such as in restaurants, as well as the actions of consumers in the home. In this review, we will discuss the occurrence of these processing contaminants, factors impacting their production, the exposure of consumers and the measures taken by regulators. We will also describe the strategies used to reduce the risk of their formation in wheat products, focusing in detail on the role of genetics and agronomy.

## 2. Acrylamide

### 2.1. Acrylamide Toxicity and Dietary Exposure

**Figure 2:**
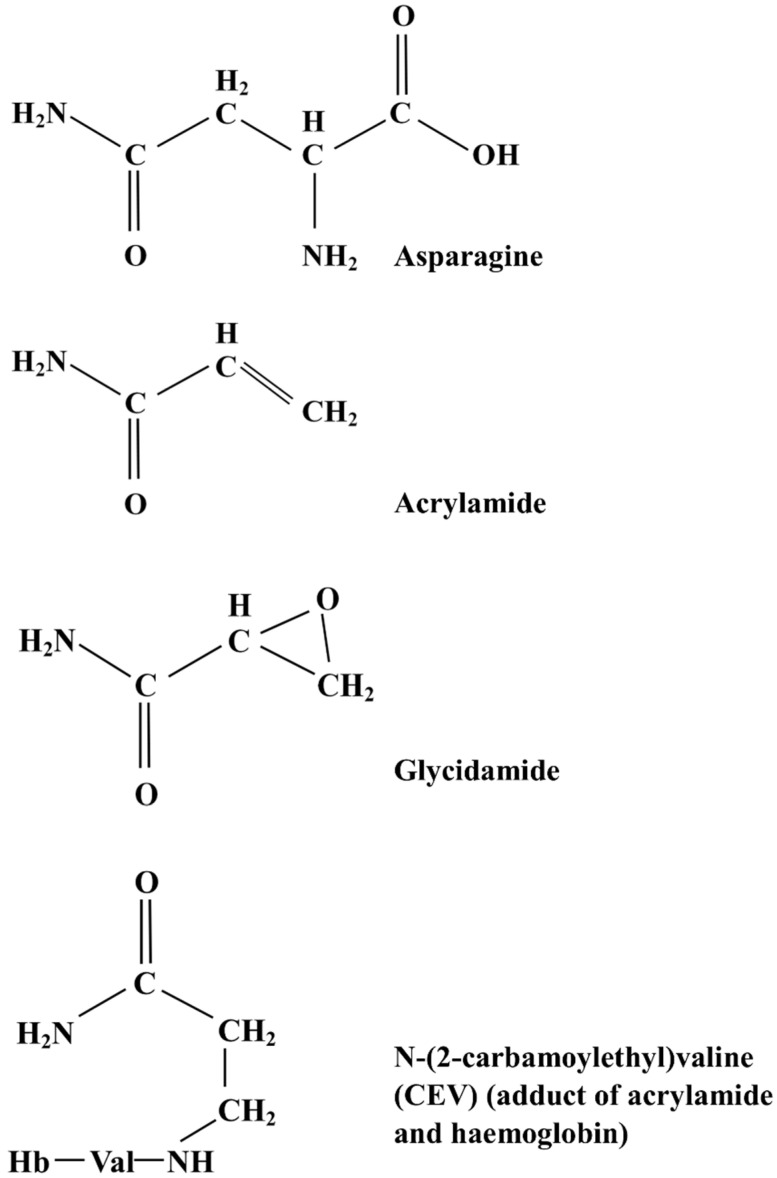
Diagrams representing the chemical structures of asparagine, acrylamide, glycidamide and the adduct, N-(2-carbamoylethyl)valine (CEV), which forms through the reaction of acrylamide with the N-terminal valine residue of a globin chain of haemoglobin (Hb).

Acrylamide (Figure 2) is a white, odourless, crystalline, water-soluble solid with the chemical formula C_3_H_5_NO and relative molecular mass of 71.08. In its monomeric form, it is regarded as a hazardous chemical: in the USA, for example, it is classified as an extremely hazardous substance under the Emergency Planning and Community Right-to-Know Act. Acrylamide is a potent neurotoxin affects male reproduction, causes birth defects and is genotoxic and carcinogenic in laboratory animals [6]. It is classified as a Group 2a carcinogen (probably carcinogenic to humans) by the International Agency for Research on Cancer (IARC) [7].

Acrylamide is metabolised to glycidamide (C_3_H_5_NO_2_) (Figure 2), and it may actually be glycidamide that is responsible for the genotoxic and carcinogenic effects attributed to acrylamide exposure. Both acrylamide and glycidamide form adducts with haemoglobin, and the detection of these adducts is the favoured method for obtaining quantitative measurements of acrylamide exposure [6]. Both also form adducts with DNA in vitro, but only glycidamide-DNA adducts have been found in human or animal tissues. These may also be used as markers for acrylamide exposure. The primary detoxification and excretion route for both acrylamide and glycidamide involves a reaction with glutathione and the conversion of the resulting adducts to mercapturic acids in the liver. These mercapturic acids are excreted, and their presence in urine represents another marker for acrylamide exposure.

The United Nations Food and Agriculture Organization (FAO) and World Health Organization (WHO) Joint Expert Committee on Food Additives (JECFA) issued reports on acrylamide exposure in 2006 and 2011 [8,9], calculating the average dietary exposure for the general population to be 1 µg per kg bodyweight per day, with consumers in the high percentile (i.e., those eating the most acrylamide-containing foods) exposed to 4 µg per kg bodyweight per day. The European Food Safety Authority (EFSA) has also provided estimates of the exposure levels of Europeans to dietary acrylamide [6], calculating a mean of 0.31 to 1.1 μg per kg bodyweight per day for adults, 0.43 to 1.4 μg per kg bodyweight per day for adolescents (11–17 years old), 0.70 to 2.05 μg per kg bodyweight per day for children (3–10 years) and 1.2 to 2.4 μg per kg bodyweight per day for toddlers (1–3 years). These figures are slightly lower than the JECFA estimate.

Toxicological studies with acrylamide have been conducted in a range of animal species, and a meta-analysis of the results of these studies was published by the European Food Safety Authority (EFSA)’s Scientific Panel on Contaminants in the Food Chain (CONTAM) in 2015 [6]. Toxicological studies use high doses of the chemical being tested so that statistically significant effects can be demonstrated, and the doses of acrylamide used are typically measured in mg per kg body weight, i.e., orders of magnitude higher than the estimates of human exposure arising from acrylamide in the diet. Nevertheless, in 2006, JECFA issued an opinion on the risks posed by dietary acrylamide [8], stating that the margins of exposure (MOEs) indicated a health concern due to acrylamide’s genotoxic and carcinogenic properties. The CONTAM Panel report of 2015 also concluded that the MOEs for acrylamide indicated ‘a concern for neoplastic effects’ [6] (the MOE is defined by EFSA as the ratio of the level at which a small but measurable effect is observed to the estimated exposure dose), essentially reaffirming the opinion issued by JECFA almost a decade earlier. However, the CONTAM Panel did not consider exposure from the diet to be sufficient to cause neurological, reproductive or developmental effects.

The CONTAM Panel [6] also considered the results of a wide range of epidemiological studies, including 36 papers investigating associations between dietary acrylamide intake and cancer risk. The Panel concluded that the results of epidemiological studies had not been consistent enough to enable firm conclusions to be drawn on whether acrylamide was a human carcinogen. Hence, the Panel’s opinion on the risk posed by dietary acrylamide was based solely on toxicological data.

Since the CONTAM report was published, a unique mutational ‘signature’ has been linked to acrylamide and glycidamide [10]. This signature was found in approximately one third of the 1600 tumour genomes analysed in the study, derived from 19 human tumour types from 14 organs. This looks alarming, but it should be noted that the study did not provide evidence that the mutations were responsible for the cancers. Nevertheless, the mutations in 184 liver tumour samples and 217 tumours of 15 other cancer types were considered to have arisen from dietary exposure to acrylamide because other mutations associated with the effects of tobacco smoke, the other major source of acrylamide exposure, were not present. On the other hand, Eisenbrand [11] reviewed the evidence for a genotoxic mode of action for acrylamide/glycidamide and found that ‘compelling evidence is lacking’, concluding that any genotoxicity of acrylamide occurred at doses that were irrelevant to dietary exposure. However, this analysis does not seem to have swayed the opinion of the CONTAM Panel or affected the European Commission’s risk management measures (Section 2.3).

### 2.2. Formation

Acrylamide is formed in foods by the thermal degradation of free (soluble, non-protein) asparagine in the presence of reducing sugars (principally glucose, fructose and maltose) in the Maillard reaction (Figure 1) [12,13,14]. The Maillard reaction, which is named after the French scientist Louis-Camille Maillard, who described it in 1912, is a complex chain of non-enzymic chemical reactions taking place between free amino acids and reducing sugars. The Maillard reaction can occur at room or even lower temperatures, but the rate is extremely slow and it is only at high temperatures (>120 °C) that it becomes relevant to food processing. It was an important reaction for the food industry long before acrylamide was discovered in food because it is responsible for the colours, flavours and aromas that are associated with fried, baked, roasted and toasted foods and are demanded by consumers. There are numerous reviews available describing the Maillard reaction [5,15,16,17,18,19,20,21] and we will not go into detail here. In brief, it involves sugar amine condensation, Amadori or Heyns rearrangement, the degradation and fragmentation of sugars as well as free amino acids and, in the last steps of the reaction, the condensation of aldols and aldehyde-amines and the formation of heterocyclic nitrogen compounds. It is when free asparagine participates in these later stages of the reaction, undergoing decarboxylation and deamination, that acrylamide is formed [12,13], with the entire carbon skeleton of the acrylamide that forms deriving from asparagine (Figure 2).

Another route for acrylamide formation from asparagine involves 3-aminopropionamide (3-APA) as a transient intermediate, with deamination of 3-APA resulting in acrylamide formation even in the absence of reducing sugars/catalysts [22]. Asparagine may also react directly with reducing sugars to form acrylamide [23]. Acrolein, acrylic acid and wheat gluten have also been postulated to be additional precursors for acrylamide [24,25].

The Maillard reaction is complex, multi-step and affected by many factors, including temperature, pH, water activity, and the presence of catalysts. Higher temperatures and alkaline conditions, for example, promote the Maillard reaction, whereas acidic conditions inhibit it, while the presence of metal ions such as copper, iron, or manganese can act as catalysts and accelerate the reaction [19]. Since the discovery of acrylamide in food in 2002, food manufacturers have sought to exploit these factors to reduce the concentration of acrylamide in their products. They have also improved the control of cooking temperature and duration, and of moisture levels in the finished product, and for some products, applied quality control based on colour. Additionally, for some products, food manufacturers have used asparaginase to reduce asparagine content as far as possible before baking [26]. These methods are more effective in some products than in others and also risk having a negative impact on product quality or the characteristics that define brands and set them apart from the competing products on the shop shelf. They have been compiled in a ‘Toolbox’ produced by FoodDrinkEurope, the latest update of which was published in 2019 [27], and are reviewed in [28].

### 2.3. Risk Management Measures (Regulations) and the Challenge of Regulatory Compliance

The amount of acrylamide present in a food sample can be measured using either gas or liquid chromatography coupled with mass spectrometry (GC-MS or LC-MS/MS). In the GC-MS method, the acrylamide has to be brominated to produce 2,3-dibromopropionamide. This is converted to 2-bromopropenamide by dehydrobromination with triethylamine, and 2-bromopropenamide is the compound that is actually loaded into the GC-MS. This makes the GC-MS method considerably more laborious than the LC-MS/MS method, which requires no prior derivatisation, and the LC-MS/MS method has become more popular in the last decade or so as the equipment has become more widely available. However, the Comité Européen de Normalisation (European Committee for Standardisation) (CEN) has developed standard methods for both (EN 16618:2015 and FprCEN/TS 17083).

The European Union set Indicative Values for different food types in 2011 [29] and reduced them in 2013 [30] (Table 1). Indicative Values were described as ‘triggers for investigation’ by the European Commission, although they were often interpreted as safety limits by consumer groups and the general media. In 2017, Regulation (EU) 2017/2158 [31] replaced Indicative Values with Benchmark Levels (Table 1). These were lower than the corresponding Indicative Values for most products and were described as ‘performance indicators’. It is unlikely that consumers understand the difference between ‘triggers for investigation’ and ‘performance indicators’, and EFSA’s data on acrylamide concentrations in food showed that the levels had been flat for most products at least since 2011, so setting the Benchmark Levels lower than the Indicative Values just meant that there were more apparent ‘failures’. Data in the public domain on acrylamide levels in cereal products are relatively sparse, but a recent study in Spain found that 15% of breakfast cereals contained acrylamide above the Benchmark Level [32]. It is alarming for the food industry and its supply chain, therefore, that the European Commission is currently considering reducing Benchmark Levels further and imposing Maximum Levels for some foods, including all of the major cereal products [33]. It would, of course, be illegal to sell a product containing acrylamide above the Maximum Level. The Benchmark and Maximum Levels that are under discussion are given in Table 1, but it should be noted that no final decision has been taken on these at the time of writing.

At the time that Regulation (EU) 2017/2158 came into force, the UK was part of the EU, and the Regulation rolled over into UK law when the UK left the EU in 2020. The EU is the UK’s largest trading partner for both wheat grain and wheat-based food products, so any developments in the EU will affect UK farmers and food businesses. What is not clear is whether the UK will follow the EU’s lead in setting Maximum Levels for acrylamide. UK food businesses and their suppliers, therefore, face uncertainty over whether the EU is about to impose Maximum Levels for acrylamide, what those levels will be, and whether Maximum Levels will also be applied in the UK. This uncertainty makes it very difficult to make the preparations necessary to ensure regulatory compliance.

No other regulatory authorities around the world have gone as far as the EU in developing risk management measures for acrylamide. The State of California did file and win a lawsuit in 2005 against a number of potato crisp (US chip) and French fry manufacturers for selling products without a Proposition 65 warning on them. Proposition 65 requires businesses in California to post warnings of any chemical in their products that may cause cancer. The lawsuit resulted in the manufacturers having to pay fines and commit to cutting the level of acrylamide in their products by between 20 and 87%. In 2010, an NGO, the Council for Education and Research on Toxics (CERT), brought a similar lawsuit under Proposition 65 against more than 90 companies producing or selling coffee. At the Federal level in the USA, however, the Food and Drug Administration (FDA) has gone no further than issuing an ‘action plan’ [34].

In Canada, acrylamide has been added to the list of chemicals in the government’s Chemicals Management Plan, and Health Canada has been working with the food industry to develop and share acrylamide reduction methods. It has also implemented an acrylamide monitoring programme and discussed the possibility of setting ‘reduction targets’. However, to date, no targets or limits have been set. Food authorities in Japan and Hong Kong and Food Standards Australia New Zealand (FSANZ) have also issued advice on acrylamide reduction without going so far as to introduce targets or limits.

### 2.4. Acrylamide in Wheat Products and the Contribution of Wheat Products to Dietary Exposure

**Figure 3:**
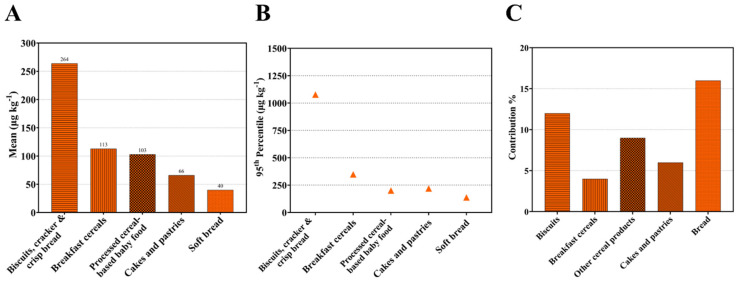
Acrylamide in cereal products in the European Union. (**A**). Mean (µg kg^−1^) and (**B**). 95th percentile (µg kg^−1^) acrylamide levels in selected cereal-based food groups or products. (**C**). Contribution of different cereal-based foods to dietary acrylamide exposure for adults. Plotted using data from [6].

Data on the presence of acrylamide in food in the European Union have been collected since 2003 and have been extremely important in informing the development of the European Commission’s risk management measures. Data on cereal products from the 2015 CONTAM Panel report [6] are shown graphically in Figure 3A (mean) and Figure 3B (95th percentile), with the highest levels in biscuits and breakfast cereals and the lowest in bread. The report also included estimates of the contribution of different foods to dietary intake in different European Union Member States, summarising the data from 16 studies in the form of a table giving the number of studies that had placed the contribution of each food type with a range of 0–5%, 5–10%, 10–25%, 25–50% and >50%. Estimates from those data of the contribution of different cereal food types to dietary acrylamide exposure in the European Union are shown graphically in Figure 3C. Bread is the highest contributor to intake despite containing relatively low concentrations of acrylamide because so much of it is consumed, and a significant proportion of it is toasted before consumption.

### 2.5. Crop Management Measures to Reduce the Acrylamide-Forming Potential of Wheat Grain

Many studies have shown the level of acrylamide formation in wheat-derived food products to depend upon the concentration of free asparagine in the grain [35,36,37,38]. Asparagine is an amino acid amide that has a molecular mass of 132.12 and an N:C ratio of 2:4 (Figure 2). It is also relatively unreactive, is a substrate for only a few enzymatic reactions and has little net charge under physiological conditions. These factors make it an ideal molecule for nitrogen storage and transport. Not surprisingly, therefore, it accumulates in plants at some developmental stages, including senescence and seed germination, and in response to various stresses, such as drought or salt stress (osmotic stress), mineral deficiency, toxic metals or pathogen attack [39]. The use of free asparagine as a nitrogen storage and transport molecule means that an asparaginase activity is required to remobilise the nitrogen when required, and asparaginase is present in many plant tissues, including wheat seeds [40]. Research on methods to reduce the free asparagine content of wheat grain has included the effects of fertilisation and disease control, varietal comparisons and genetic approaches to provide wheat breeders with the resources and knowledge to produce low asparagine varieties.

#### 2.5.1. Fertilisation

Given the potential role of free asparagine in nitrogen transport and storage, it is no surprise that its concentration in wheat grain rises in response to increased nitrogen availability. Increased nitrogen supply was shown to cause a rise in free asparagine concentration in barley grain as long ago as 1980 [41]. This was considered to be of largely academic interest until free asparagine was shown to be a precursor for acrylamide formation. Subsequent studies showed that increased nitrogen fertilisation of wheat promoted free asparagine accumulation, with a concomitant effect on acrylamide formation in bread [42,43]. The effect of nitrogen availability, however, was found to be dwarfed by that of sulphur deficiency. Increases of up to 30-fold in free asparagine concentration were measured in response to severe sulphur deficiency imposed in a pot experiment, for example [35], and a series of pot- and field-based studies have confirmed the effect [36,37,44,45,46]. Indeed, sulphur deficiency is by far the most important factor affecting the concentration of free asparagine in wheat grain, and varieties with low free asparagine under sulphur-fed conditions tend to be more affected, meaning that any attempt to rank varieties according to free asparagine concentration in grain breaks down under conditions of sulphur deficiency [45].

Analyses of the contrasting gene expression patterns in the developing seeds of wheat grown with and without sulphur supplied showed gene expression in the embryo to be the determining factor for asparagine accumulation in the grain, with increased expression of asparagine synthetase-2 (*TaASN2*) and glutamine synthetase in the embryo in response to sulphur deficiency and a decrease in asparaginase gene expression [40]. The signalling networks responsible for these changes in gene expression have not yet been elucidated, but genes encoding regulatory protein kinases, sucrose nonfermenting-1-related protein kinase-1 (SnRK1) and general control nonderepressible-2 (GCN2), also responded to sulphur deficiency [40,47]. Both of these have been implicated in regulating asparagine synthetase gene expression, possibly in the case of SnRK1 via bZIP transcription factors that contain SnRK1 target sites, including Opaque2/bZIP9, SPA/bZIP25 and BLZ1/OHP1/bZIP63, all of which are expressed in wheat seeds [40,47].

**Figure 4:**
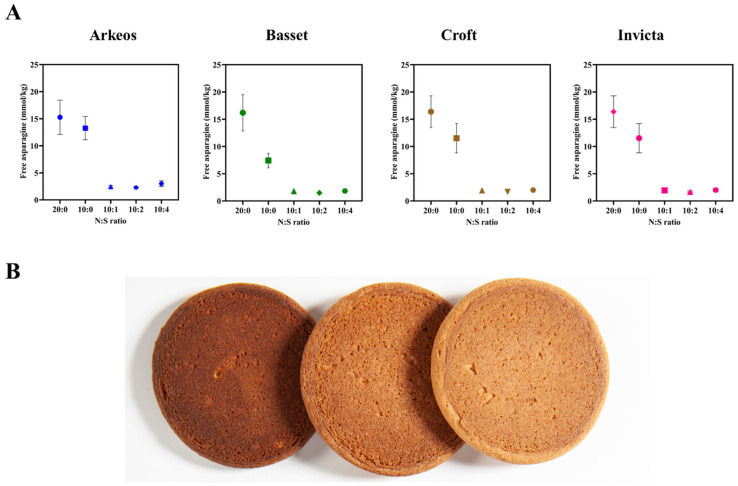
Effects of the nitrogen to sulphur fertilisation ratio on free asparagine concentration. (**A**). Graphs representing the effect of different nitrogen to sulphur ratios on free asparagine content (mmol kg^−1^) in the grain of four different wheat varieties. Each point represents the mean value from two field trials carried out in years 2019/2020 and 2020/2021 at two different locations. (**B**). Biscuits made from the Basset variety grown with different nitrogen to sulphur ratio treatments. N:S from left to right: 20:0, 10:0 and 10:1. Data from [38]. Image produced by Graham Shephard (Rothamsted Research) and kindly provided by Joseph Oddy.

These results are consistent with the plant using free asparagine as a nitrogen store in the grain and directing more nitrogen into free asparagine accumulation when sulphur is not available. This may, at least in part, be because it has insufficient sulphur to make sulphur-rich seed storage proteins [48]. This hypothesis led to an investigation of the importance of the N:S ratio in fertiliser application [38]. That study found the effect of the N:S ratio to be significant (*F* probability < 0.001) over two years of field trials. There was also an interaction between the N:S ratio and variety, meaning that the varieties in the trials responded differently to the treatment [38]. Figure 4A shows the responses (asparagine content) of four varieties in the trial, and Figure 4B shows the darker colour of biscuits cooked with flour from the low sulphur plots. Biscuits made with flour from plots fertilised with 100 kg ha^−1^ nitrogen and 10 kg ha^−1^ sulphur contained a mean of 488 μg kg^−1^ acrylamide, while biscuits from the 100 kg ha^−1^ nitrogen, no sulphur plots, contained 6114 μg kg^−1^ and the 200 kg ha^−1^ nitrogen, no sulphur plots an astonishing 13,523 μg kg^−1^ acrylamide. Biscuit acrylamide correlated with the free asparagine content of the grain and with biscuit colour (top surface hue angle), with R^2^ values from linear models of 89% and 91%, respectively (*p* < 0.001). The acrylamide figures show just how much can form in foods if the raw material has a lot of free asparagine in it, and the importance for manufacturers of not allowing wheat grown with insufficient sulphur availability into their processing lines. They also showed very nicely the interaction between nitrogen and sulphur. It was concluded that a nitrogen to sulphur ratio of 10:1 was required and sufficient to prevent large increases in the accumulation of free asparagine. Ensuring that ‘good agricultural practices’ are followed on fertilisation, to ‘maintain balanced sulphur levels in the soil and to ensure correct nitrogen application’ are also included in Regulation (EU) 2017/2158 [31].

The study also investigated the effect of withholding two other important minerals for crop growth and health, potassium and phosphorus, but found that deficiencies in these minerals did not cause increases in free asparagine concentration when sulphur was applied, so the response is specific to sulphur availability [38].

#### 2.5.2. Disease Control

**Figure 5:**
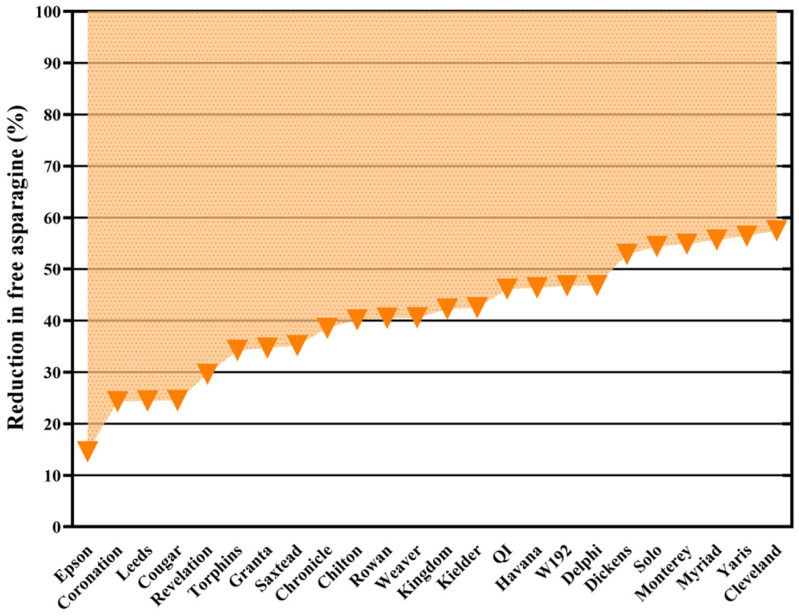
Graph representing the impact of disease control on free asparagine content in the grain of 24 winter wheat varieties. Each triangle represents the percent reduction in free asparagine content in grain from plots treated with fungicide compared with grain from untreated plots. The fungicide treatment was given from growth stages 30 to 65. The main diseases observed in the untreated plots were *Septoria tritici*, yellow rust and brown rust. Drawn using data from [51].

Asparagine concentration in plant tissues is also sensitive to biotic stress [39,49], possibly due to asparagine’s involvement in the remobilisation of nitrogen and/or ammonia detoxification during pathogen attack [50]. A field-based study in 2009 showed that fungicide treatment of three wheat varieties reduced free asparagine accumulation [43]. A larger study was then performed with 24 varieties of wheat grown in a field trial in the UK in 2011–2012, with adjacent plots treated in an identical fashion, except that fungicide was applied to one plot and not the other, the lack of fungicide resulting in visible infection by *Septoria tritici*, yellow rust and brown rust [51]. Both free asparagine concentration and acrylamide formation in flour (heated for 20 min at 180 °C) reduced in response to fungicide treatment, and the reductions in free asparagine concentration are shown in Figure 5. These studies showed disease control by fungicide application to be an important crop management measure for mitigating the problem of acrylamide formation in wheat products, and EC Regulation (EU) 2017/2158 [31] states that ‘application of good practices on crop protection measures to prevent fungal infection’ must be ensured. It is ironic, therefore, that the European Commission has recently made crop protection more difficult for farmers by withdrawing or refusing to renew the licences for some fungicides, giving the impression that different offices within the Commission do not communicate with each other.

**Figure 6:**
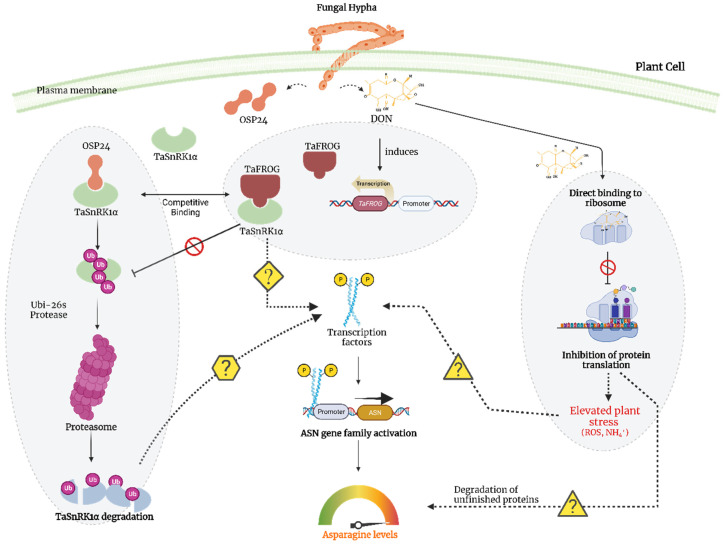
Working model of the signalling hub linking pathogen (*Fusarium graminearum*) infection with asparagine synthetase gene expression in wheat. DON (deoxynivalenol) binds to ribosomes and inhibits protein synthesis, leading to the accumulation of reactive oxygen species (ROS) and free ammonia (NH_4_^+^). DON also induces expression of the *TaFROG* gene, and TaFROG competes with a fungal protein, OSP24, for binding to TaSnRK1α, thereby safeguarding TaSnRK1α from degradation. However, the precise mechanism through which this signalling pathway ultimately activates the asparagine synthetase gene family and the transcription factors involved remain unknown. This model is based on an adaptation from [52,55]. Created with BioRender.com.

The interplay between the fungal and plant molecular factors involved in this aspect of plant–pathogen interactions is fascinating, even with much of the system still to be elucidated. There is evidence that SnRK1, which was discussed above in the context of the sulphur response, also plays a central role in co-ordinating pathogen defence mechanisms and activation of adaptive responses, including the increase in asparagine synthesis. Perochon et al. [52], for example, reported that SnRK1 is involved in the response of wheat to infection by *Fusarium graminearum* (*Fg*), the main causal agent of Fusarium head blight disease (FHB, also known as ‘scab’). This disease reduces yield and grain quality and contaminates grain with mycotoxins, most commonly deoxynivalenol (DON), a toxic sesquiterpenoid. DON treatment of wheat has been shown to increase the levels of free asparagine, glutamine and aspartate in the grain [49,53]. Under *Fg* infection, the expression of a gene encoding a wheat orphan protein, TaFROG, is upregulated, and the interaction of TaFROG with SnRK1 enhances FHB and DON resistance [54]. In direct opposition to the action of TaFROG, an *Fg*-secreted effector protein, OSP24, interacts with SnRK1 and promotes SnRK1 degradation by the ubiquitin-26S proteasome. TaFROG competes with OSP24 for binding with SnRK1, thereby protecting it from degradation [55]. Our current working model of these interactions is given in Figure 6.

### 2.6. Genetic Approaches to Reducing the Acrylamide-Forming Potential of Wheat Grain

Free asparagine concentration has been shown to vary among wheat varieties in several studies [35,36,37,45,51,56,57]. Curtis et al. [45], for example, reported a range of free asparagine concentrations from 0.708 to 11.29 mmol kg^−1^ in different varieties of sulphur-fed wheat in a 2012–2013 field trial in the UK, while Tafuri et al. [57] reported a much tighter range of 0.55 to 2.84 mmol kg^−1^ in 54 Italian bread-making varieties over two years of field trials. It is clear from these studies that genetic factors (G) play a part in determining free asparagine concentration, suggesting that it might be possible to reduce acrylamide formation in wheat products by selecting low asparagine varieties and that free asparagine concentration in wheat grain may be amenable to breeding. However, there are some obstacles that make both of those aspirations difficult to achieve. The first is the impact of environmental factors (E), within which we would include crop management for optimum fertilisation and good disease control. Neither sulphur deficiency nor disease affects different varieties to the same extent; if anything, they cause greater increases in free asparagine in otherwise low asparagine varieties, meaning that varietal rankings break down; i.e., there is a large G × E component to the variance.

Despite the effects of E and G × E, Curtis et al. [45] did identify eight varieties that were consistently low in free asparagine in the grain. However, food businesses who wish to purchase low asparagine wheat, at least in the UK, face another obstacle in that the turnover in wheat varieties is so rapid that by the time the free asparagine concentration of the grain of a new variety has been measured over enough seasons to give an accurate assessment, the variety is no longer on the market. The solution to this problem would be for breeders to measure free asparagine concentration during variety development so that the information could be provided when the variety was launched. To date, however, this has not been done, and in our view, this needs to change.

The food industry would undoubtedly also like to see more progress from breeders in producing low-asparagine wheat varieties. However, to our knowledge, it remains a low priority for breeders, who continue to chase their traditional targets of yield, protein content and disease resistance. We may see more action on asparagine content if the regulatory situation in the EU changes in the expected way (Table 1) and the pressure from the food industry intensifies, or if readily-usable tools and resources can be developed to make the task easier for breeders.

#### 2.6.1. Identifying Quantitative Trait Loci (QTL) That Control Free Asparagine Accumulation in Wheat Grain

Knowledge about the genetic architecture or QTL associated with the free asparagine content of wheat grain could enable the development of genomic tools to assist breeders in the selection of low asparagine varieties in the early generations of a breeding programme. However, to date, the search for consistent QTL has been frustrating. In a study conducted by Emebiri in 2014 [58], for example, 92 different wheat varieties were examined, with a wide range of free asparagine concentrations. Through a genome-wide scan for single-nucleotide polymorphism (SNP) markers associated with this variance, nine SNPs were identified on chromosome 5A. These SNPs accounted for 14–24% of the observed variation in free asparagine concentration and could be potential candidates for further investigations aimed at developing molecular markers. However, the study concluded that breeding cultivars with naturally low levels of grain asparagine would pose a challenge, as the heritability of the trait was only 32%.

Subsequently, in 2018, Peng et al. [59] conducted a study on 182 bread wheat accessions, employing a single-locus genome-wide association study (GWAS) with a mixed linear model (MLM) and six multi-locus GWAS models. They reported that an aminopeptidase on chromosome 4B served as an underlying factor in controlling free asparagine content in wheat. Further, in 2018, Rapp et al. [60] mapped eight putative QTL explaining 78.5% of the variance for free asparagine content among 149 wheat varieties, with the QTL accounting for the highest variance (18.4%) on chromosome 7B. However, the QTL detected in this study were different from those reported by Emebiri [58], presumably arising from the strong impact of environmental factors and signifying that the QTL being identified were based on phenotypic data specific to the local environment.

In 2023, Oddy et al. conducted another study [61], this time to examine the genetic architecture underpinning the variance in levels of free asparagine in a doubled haploid mapping population produced from a cross between varieties Claire and Robigus. These varieties have been extensively used as parent lines in soft wheat breeding in the UK, and have been shown to have contrasting concentrations of free asparagine in the grain, with the concentration in Claire lower and more consistent than that in Robigus [37]. A QTL for free asparagine content in grain was identified on chromosome 4B, located approximately 60 Mbp away from a QTL reported by Peng et al. [59], the first time that two studies had identified QTL that might overlap. However, this QTL was also affected by environmental factors, accounting for only 2.6% of the variance in a 2017–2018 field trial of the population, but 14.8% of the variance in a 2018–2019 field trial when free asparagine concentrations were higher [61]. Even so, the Robigus allele was associated with higher free asparagine concentrations in both years.

**Figure 7:**
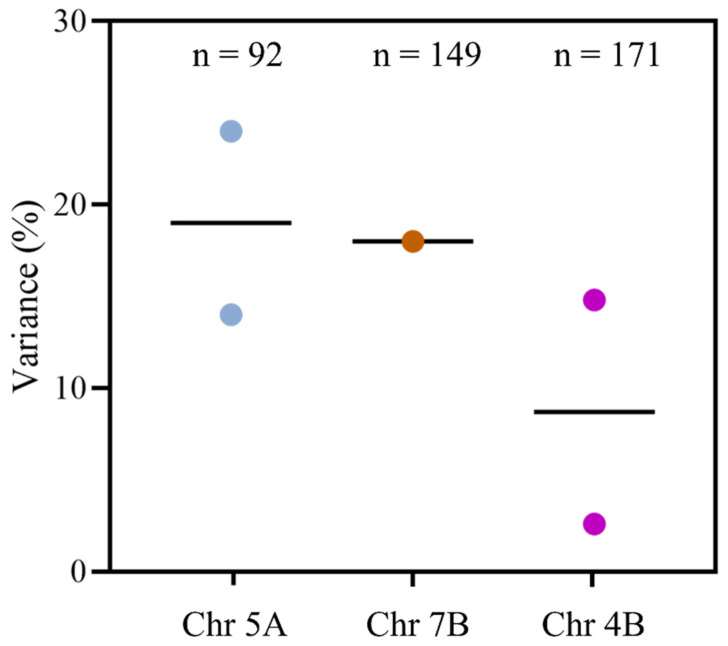
Graph illustrating the maximum amount of variance in free asparagine concentration in the grain that can be attributed to quantitative trait loci (QTL) on chromosomes 5A, 7B and 4B in different varieties of wheat. The QTL on chromosome 5A was responsible for explaining 14–24% of the variance, while those on chromosomes 7B and 4B explained 18.4% and 2.6–14.8% of the variance, respectively. The black lines in the plots indicate the average percentage of variance observed. Plotted using data from [58,59,61].

The contributions of the different QTL to the variance in free asparagine concentrations between varieties are shown graphically in Figure 7. The overall conclusion to be drawn from these studies is that ‘traditional’ breeding alone probably has limited scope for reducing free asparagine concentration in wheat grain.

#### 2.6.2. The Asparagine Synthetase Gene Family of Wheat and Characterisation of a Natural Genetic Event Associated with Reduced Free Asparagine Concentration in Wheat Grain

Asparagine is made via the ATP-dependent transfer of an amino group from glutamine to aspartate to generate glutamate and asparagine. The reaction is catalysed by the enzyme asparagine synthetase and wheat, like all the members of the Triticeae tribe, has five asparagine synthetase genes: *TaASN1*, *TaASN2*, *TaASN3.1*, *TaASN3.2* and *TaASN4* [62,63]. Of these, *TaASN2* is most highly expressed in the grain, while it is not expressed elsewhere in the plant. The closely related gene, *TaASN1*, is the next most highly expressed gene in the grain but is also expressed elsewhere [64]. For both genes, expression in the embryo is much higher (approximately 10-fold) than in the endosperm [64]. This was confirmed in an analysis of RNA-seq data for developing grains, with *TaASN2* gene expression in the embryo increasing in response to sulphur deficiency, in line with the observed increase in free asparagine concentration in the grain [40]. That study also showed the A genome homeologue, *TaASN-A2*, to be much more highly expressed than *TaASN-D2*, while *TaASN-B2* expression was not detectable at all in those genotypes [40].

**Figure 8:**
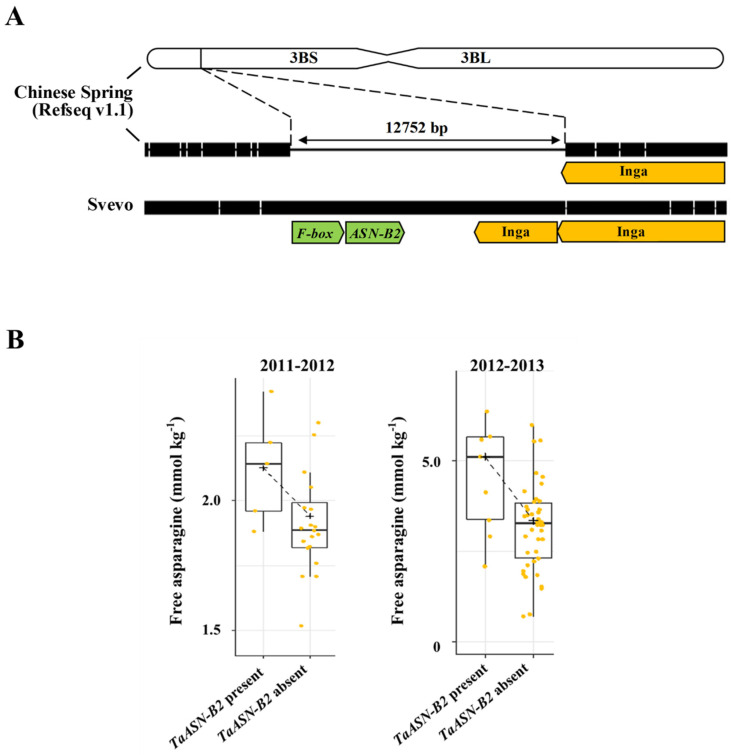
(**A**). Representation of the region of chromosome 3B of wheat variety Chinese Spring in which there is a natural deletion of ~13 kb, and the corresponding region in variety Svevo, in which the deletion has not occurred and the *TaASN-B2* gene is still present. (**B**). Graph showing the impact of the presence/absence of the *TaASN-B2* gene on free asparagine content in the grain of 50 varieties of winter wheat in two consecutive field trials (2011–2012; 2012–2013). Adapted from [65].

Analyses of wheat genome data showed all of the *TaASN* genes to be present as a single copy per genome, with *TaASN1* on chromosome 5, *TaASN2* on chromosome 3, *TaASN3.1* and *TaASN3.2* on chromosome 1 and *TaASN4* on chromosome 4 [62,63]. These analyses also showed that variety Chinese Spring lacked a *TaASN2* gene on the B genome (*TaASN-B2*) as a result of a natural deletion of almost 13 kb (Figure 8A). A subsequent study by Oddy et al. [65] determined the presence and absence of the *TaASN-B2* gene across a range of UK and global common wheat varieties and related species. The study showed that the deletion encompassing the *TaASN-B2* gene was present in some wild emmer wheat genotypes and most, but not all, cultivated varieties. Notably, there was a trend for free asparagine concentrations in field-produced grain to be lower in varieties lacking *TaASN-B2* (Figure 8B), although the effect was lost when free asparagine accumulated to very high concentrations as a result of sulphur deficiency [65].

These results suggest that selecting wheat genotypes lacking the *TaASN-B2* gene may be a simple and rapid way for breeders to reduce free asparagine concentrations in commercial wheat grain. However, most varieties already lack the gene, across all milling types, which would explain why the null allele has not been identified in QTL studies: both Claire and Robigus, for example, lack a *TaASN-B2* gene, so the effect of the presence/absence of the gene would not have been detected in the analysis of the Claire × Robigus mapping population [61]. This limits the scope for breeders to exploit this natural genetic event. On the other hand, it is something that breeders could be doing already, but to our knowledge, they are not.

#### 2.6.3. The Application of Genome Editing and Chemical Mutagenesis to Reducing Free Asparagine Concentrations in Wheat Grain

The discovery that *TaASN2* is expressed grain-specifically and is by far the most highly expressed asparagine synthetase gene in the grain, makes it an obvious target for genetic interventions, and in 2021, Raffan et al. [66] reported the generation of bread wheat cv Cadenza genotypes in which *TaASN2* had been ‘knocked out’ using CRISPR-Cas9. The editing was achieved by introducing a 4-gRNA polycistronic gene into wheat embryos along with a *Cas9* gene and a marker gene (*Bar*). Free asparagine concentration in plants in which all six *TaASN2* alleles were knocked out was 43–57% of the wildtype over two generations [66], and in plants that were subsequently found to be edited in *TaASN1* as well as *TaASN2* (i.e., *TaASN1/TaASN2* total knockouts) [67] was 9–48% of the wildtype [65]. In plants containing edits only in the A genome *TaASN2* alleles, the concentration of free asparagine was 56–68% of wildtype [66].

The fact that *TaASN2* is present as a single copy gene means that it has also been possible to identify plants carrying mutations in the A, B or D genome genes within mutant wheat populations (known as TILLING populations) of cv. Cadenza and cv. Kronos (a tetraploid, genomes AB) produced by ethyl methanesulphonate treatment [68]. Alarcón-Reverte et al. [69] reported reductions in free asparagine concentration of 28% in A genome TILLING nulls in the Cadenza background, and 24–34% in the tetraploid Kronos background in field trials.

**Figure 9:**
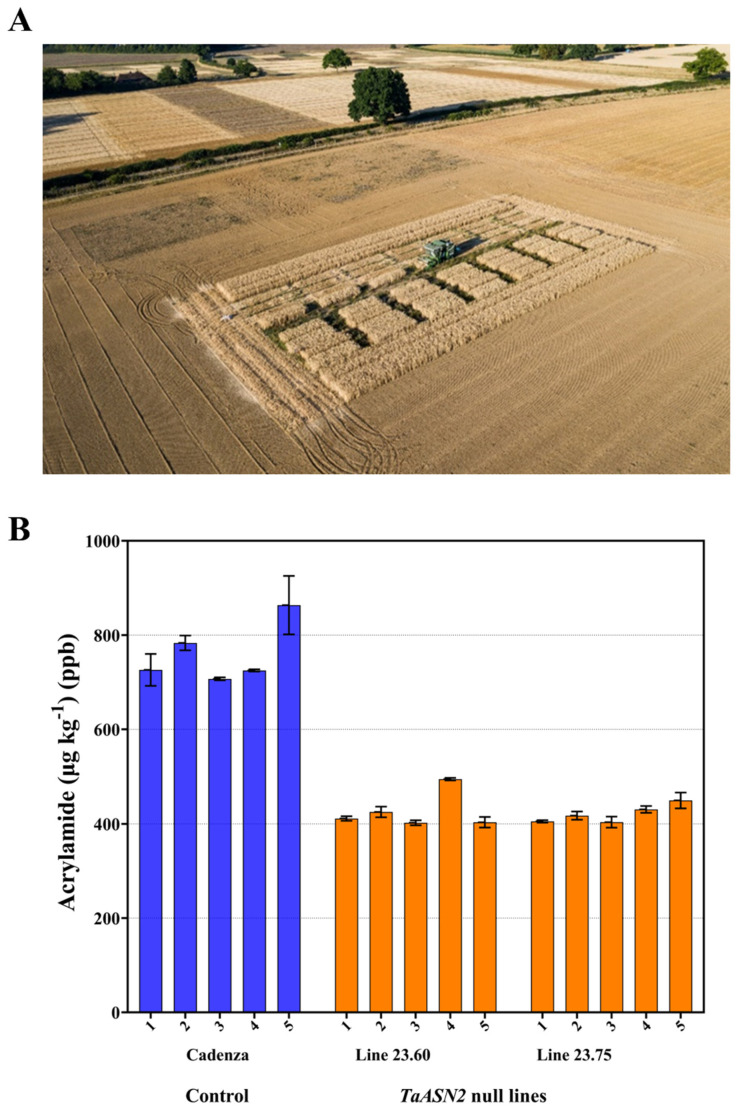
(**A**). Drone picture of Europe’s first genome-edited wheat field trial being harvested at Rothamsted, August 2022, showing the layout of 56 plots of genome-edited lines (Cadenza background), TILLING lines (Claire background) and controls in a randomised block design, with a surrounding pollen barrier. (**B**). Graphs representing the acrylamide content of heated flour (160 °C for 20 min) from five individual plots each of genome-edited *TaASN2* null lines (Line 23.60 and line 23.75) and the Cadenza control. Data from [67].

In 2021–2022, Raffan et al. carried out field trials of *TaASN2* A genome null and total null gene-edited lines (Figure 9A) [67]. Also included in the field trial were AB genome nulls derived by backcrossing an A genome null TILLING line into the cv. Claire background (cv. Claire lacks a B genome *TaASN2* gene due to the ‘natural’ deletion described in Section 2.6.2 [65]).

The mean free asparagine concentrations in the total *TaASN2* nulls were just under 50% that of the Cadenza control, while in the A genome nulls the concentration was 86% that of Cadenza [67]. The TILLING lines showed more variable responses: they did show a reduction of 20–40% compared with a TILLING control (a line that had come through the same process but did not carry mutations in *TaASN2*), but Claire was also lower than the TILLING control, so further trials will be required before firm conclusions can be drawn. The lower free asparagine concentration in the grain from the *TaASN2* total null edited lines was reflected in the amount of acrylamide that formed when flour was heated to 160 °C for 20 min, with an almost 50% decrease compared with the control. The data for individual plots of two total null lines (23.60 and 23.75) are shown graphically in Figure 9B; the means for these lines were 427 and 421 μg kg^−1^, respectively, compared with 761 μg kg^−1^ in the Cadenza control [67].

This study showed that step reductions in the free asparagine concentration and, therefore, the acrylamide-forming potential of wheat grain could be achieved using genome editing, with the reductions observed under glass maintained in the field. Importantly, while thousand grain weight was lower in the edited lines, yield was not significantly reduced; in other words, the edited lines produced more but smaller grains, although the grain size was well within the normal range for wheat. In contrast, there was a yield drag for the TILLING lines, presumably due to the background mutations introduced by the chemical treatment, making it a nice example of the advantages of the targeted versus random technique.

### 2.7. The Use of Imaging Techniques to Predict Free Asparagine Concentrations in Wheat Grain and the Anticipated Development of Free Asparagine Sensors

The use of hyperspectral imaging in plants has proven to be an efficient method for measuring plant metabolites [70] and has the potential to assist growers, millers and food processors in evaluating the quality of grains. In a study conducted by Oddy et al. [38], a Tec5 HandySpec Field spectrometer and Videometer SeedLab system were employed in the field to predict the free asparagine concentration of grain that was subsequently harvested with an average accuracy of 71%, demonstrating the effectiveness of this approach. Furthermore, a combination of multispectral, fluorescence and morphological measurements of grains after harvest could distinguish between high and low free asparagine grain with an accuracy of 86%.

The use of imaging methods to predict free asparagine concentration requires further research to confirm how consistently these prediction figures can be achieved. However, we are also aware of work being carried out by several teams on the development of on-site sensors to enable free asparagine concentration in grain to be measured ‘at the factory gate’, enabling food businesses to turn away high asparagine grain before it enters their manufacturing lines. Some of these teams claim to be in an advanced stage of development, although we are not aware of publications in the scientific literature that describe such a sensor.

The development of these systems would help food businesses to comply with regulations on acrylamide more consistently, taking away the concern that the use of grain with unexpectedly high concentrations of free asparagine could undermine their mitigation measures. For farmers, on the other hand, it raises the possibility that high-value markets could be shut off from them if the grain they produce contains too much free asparagine, and we expect farmers to look to breeders to help them produce grain that food businesses will use.

## 3. Other Processing Contaminants in Wheat Products

### 3.1. 5-Hydroxymethylfurfural (HMF)

**Figure 10:**
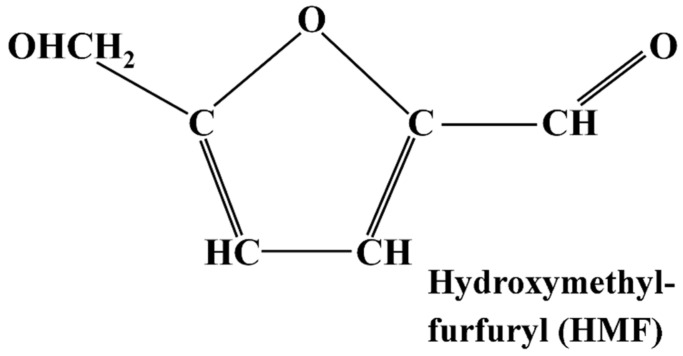
Diagram representing the structure of hydroxymethylfurfuryl (HMF).

Hydroxymethylfurfural (HMF), a furanic compound with both aldehyde and alcohol functional groups (Figure 10), has been known to be a problem in cereal products for longer than acrylamide. Like acrylamide, HMF is formed through the Maillard reaction during thermal processing (Figure 1) [71,72], but it can also be produced by the direct dehydration of fructose or caramelisation [73,74]. It is produced via an intermediate compound, 3-deoxyosone, which undergoes dehydration and cyclisation to produce HMF. HMF is thought to possess some beneficial biological properties, including antioxidant activity, the inhibition of red blood cell sickling, and the improvement of haemorheology [75]. However, in humans, HMF is metabolised to 5-hydroxymethyl-2-furoic acid (HMFA), which is excreted in urine, and 5-sulfoxymethylfurfural (SMF). SMF can form adducts with DNA or proteins, and rodent toxicology studies have indicated potential genotoxicity and carcinogenicity [76,77].

HMF is a common contaminant of dark beers [77] and is found in a variety of other food items, such as bakery products, brown sugar, caramels, breakfast cereals, malt, fruit juices, coffee, and vinegar. Among these, bread and coffee are the primary contributors to dietary intake, although dried fruits [78], caramel and vinegar can have particularly high concentrations. The potential toxicity of HMF in humans is still being investigated, but studies have indicated that high concentrations can cause cytotoxic effects and irritation to the eyes, upper respiratory tract, skin, and mucous membranes [79].

HMF can form under acidic conditions at lower temperatures [80]; however, its concentration significantly increases with higher temperatures during thermal processing or storage. Hence, HMF can serve as a valuable indicator for monitoring heating processes in food production, including pasta drying, bread baking and toasting, as well as the extrusion of cereal-based baby foods and breakfast cereals. It is sometimes used as an indicator of excessive heat treatment in biscuit manufacture, and products containing high levels of HMF are likely to contain a lot of acrylamide. HMF concentration is usually measured using high-performance liquid chromatography (HPLC). Mesías et al. [81] reported levels of 14.2–42.0 mg kg^−1^ in breakfast cereals, while Petisca et al. [82] reported levels of 3.0 mg kg^−1^ in cakes and pastries and 7.8 mg kg-1 in biscuits, with high variability between different batches of the same product. However, Ortu and Caboni [83] reported much higher concentrations in bakery products, ranging from 75–395 mg kg^−1^.

The reduction of HMF levels in food products primarily relies on the chosen processing methods and parameters. There have been no attempts that we are aware of to reduce the concentration of its precursors through genetic or agronomic approaches, for example by assessing the range of fructose and glucose concentrations in different wheat varieties or how these concentrations are affected by crop management. This is surprising because glucose and fructose levels are affected by environmental factors that could be alleviated by crop management, for example increasing in response to drought stress as the plant interconverts simple sugars and complex carbohydrates for osmotic regulation [5].

### 3.2. Polycyclic Aromatic Hydrocarbons (PAHs)

**Figure 11:**
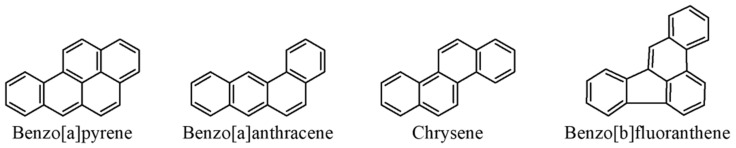
Diagrams representing the structures of the PAHs making up the so-called PAH4, the principal PAHs of cereal products.

Polycyclic aromatic hydrocarbons (PAHs) are a group of nearly 10,000 chemical compounds possessing 2–7 fused aromatic rings generated during incomplete combustion of organic compounds. Direct pyrolysis of food constituents, such as proteins and fats, can result in the formation of PAHs, and intense thermal processing methods like toasting, roasting, frying and other high-temperature cooking techniques contribute to PAH generation. PAHs have gained significant attention and recognition as priority pollutants due to their mutagenic and carcinogenic properties [84,85,86,87]. They have been included in the priority pollutant lists of several authoritative bodies, such as the Agency of Toxic Substances and Disease Register (ATSDR), the International Agency for Research on Cancer (IARC), the European Union (EU) and the United States Environmental Protection Agency (EPA). The four principal PAHs in cereal products are benzo[a]pyrene, benzo[a]anthracene, benzo[b]fluoranthene and chrysene, the so-called PAH4 (Figure 11), and the European Commission has set a tolerance limit of 1 μg kg^−1^ for PAH4 in cereal products for infants [88]. PAHs present diverse risks to human health, encompassing conditions such as asthma, breast cancer, cardiovascular disease and diminished sperm quality [89]. Additionally, PAHs can impact the prenatal development of foetuses in pregnant women. Exposure to PAHs has also been linked to impaired cognitive function in childhood and decreased birth rates [89].

**Figure 12:**
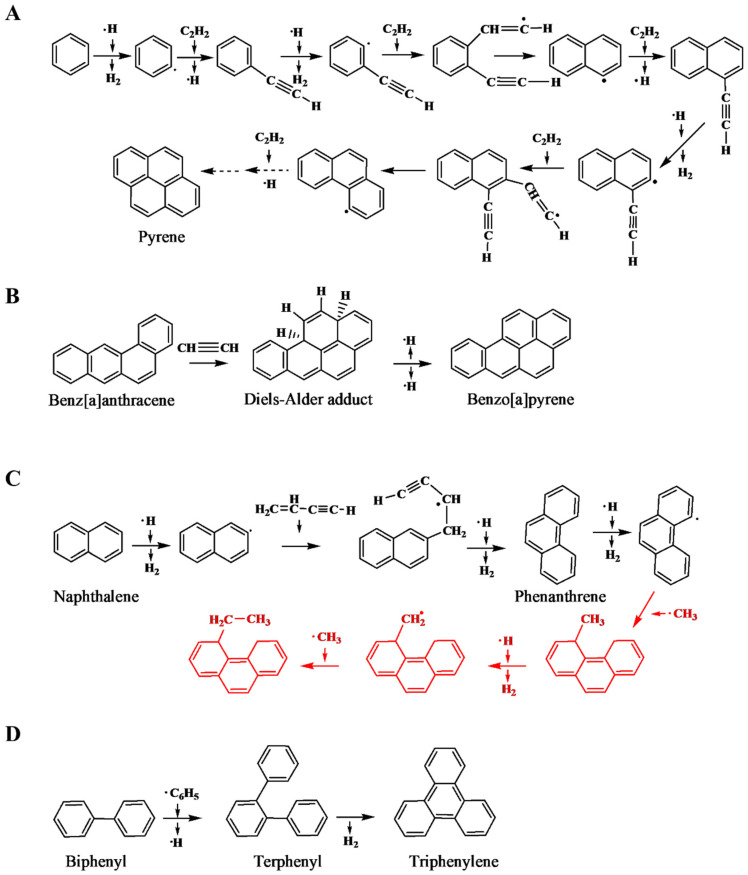
Schematic representation of structural expansion mechanisms of PAHs. (**A**). Generation of pyrene through the hydrogen abstraction and acetylene or carbon addition (HACA) process. (**B**). Creation of benzo[a]pyrene via the Diels-Alder-type reaction. (**C**). Formation of phenanthrene from naphthalene through the hydrogen abstraction vinyl acetylene addition (HAVA) mechanism, followed by the introduction of an ethyl chain onto phenanthrene using the methyl addition cyclisation (MAC) mechanism (shown in red). (**D**). Production of triphenylene from biphenyl through the phenyl-addition cyclisation (PAC) mechanism. Figure adapted from [90].

Although polycyclic aromatic hydrocarbons (PAHs) are composed of multiple aromatic rings, the initial creation of the primary aromatic ring (benzene or phenyl) is important. Multiple formation mechanisms have been proposed, involving different reactants and reaction pathways. Examples include the interaction between the vinyl radical (C_2_H_3_) and 1,3-butadiene (1,3-C_4_H_6_), followed by hydrogen removal, the reaction of diatomic carbon (C_2_) with 1,3-butadiene, and the combination of ethylene (C_2_H_4_) with cyclopentadiene (C_5_H_6_) [90]. Once the initial aromatic ring forms, subsequent structural expansion happens through diverse mechanisms, resulting in the development of polycyclic aromatic configurations. The processes of PAH creation and enlargement are mainly reliant on the source of carbon and the surrounding environmental conditions. Consequently, a comprehensive approach is imperative to comprehend these mechanisms. Four PAH formation pathways are illustrated in Figure 12.

Research on PAH exposure consistently highlights that foods, particularly those that are cereal-based, serve as the primary source of human exposure to these compounds [91,92,93]. PAHs can potentially be found in wheat products due to environmental contamination of crops or products arising from atmospheric deposition of pollutants from industrial and vehicle emissions, or formation during processing and cooking. High-temperature cooking techniques such as baking, toasting, roasting and frying can lead to the direct deposition of PAHs onto the surface of the food. Additionally, when food is exposed to smoke for flavouring or preservation purposes, PAHs present in the smoke can be deposited onto the food.

The levels can vary due to several factors, including growing conditions, storage practices and processing methods. Ahmed et al. [94], for example, conducted a study that demonstrated the influence of the fuel type used during baking on the levels of PAHs in bread and observed a range of PAH concentrations in bread baked in different types of bakeries. For instance, bread baked in mazut (heavy fuel oil)-operated bakeries had a PAH concentration of 321 μg kg^−1^, while bread baked in solid waste, solar, and electricity-operated bakeries had concentrations of 317, 158 and 26 μg kg^−1^, respectively. Rey-Salgueiro et al. [95] also observed significant effects of the toasting technique on the levels of PAHs in bread, with charcoal and flame grilling resulting in higher levels compared with electric oven and toaster methods. Wood flame toasting produced the highest levels, with up to 350 3.65 μg kg^−1^ total PAHs. In line with this, Rascón et al. [96] determined that home toasting escalated PAH levels, with the degree of increase influenced by the specific raw material used. Among different bread varieties, white bread demonstrated the highest PAH content (17.8 3.65 μg kg^−1^), closely followed by wholegrain bread (16.9 3.65 μg kg^−1^), multiseed bread (16.5 3.65 μg kg^−1^), black bread (13.0 3.65 μg kg^−1^) and sliced bread (10.3 3.65 μg kg^−1^). Ciecierska and Obiedziński [97] noted that the collective amount of 19 PAHs present in grain, flour and bran exhibited a range of 1.07 to 3.65 3.65 μg kg^−1^. The PAH content of bread varied between 1.59 and 13.6 3.65 μg kg^−1^, depending on both the specific portion of the bread analysed and the temperature employed during baking. Similarly, Chawda et al. [98] investigated total PAH concentration in Indian tandoori and tawa breads, observing a range of 113.36–211.19 3.65 μg kg^−1^ and 59.64–77.12 3.65 μg kg^−1^, respectively, representing a 71.7% difference between the two types.

Tran-Lam et al. [99] analysed PAH levels in instant noodles, fried noodles and cakes. The mean concentration in noodles was 57.2 μg kg^−1^, but this increased almost three-fold to 182.8 μg kg^−1^ when the noodles were fried. The concentration in cakes ranged from 2.32–26.92 μg kg^−1^. There has also been an analysis of PAH concentrations in Nigerian biscuits prepared using different baking techniques, fuel sources, oven conditions and ingredients, revealing a wide range of concentrations in shortcake (35.7 to 645.3 μg kg^−1^) digestives biscuits (75.9 to 490.7 μg kg^−1^) cookies (91.5 to 537 μg kg^−1^), shortbread (18.4 to 522.2 μg kg^−1^), wafers (123.5 to 393.8 μg kg^−1^), crackers (167.2 to 880 μg kg^−1^) and cabin biscuits (135.5 to 241.6 μg kg^−1^) [100]. Therefore, just like with acrylamide and HMF, the levels of PAHs in food products can be lowered by selecting effective processing methods, and this has been the focus of mitigation methods to date.

## 4. Concluding Remarks

Cooking or high-temperature processing kills potentially pathogenic micro-organisms, tenderises food, makes it palatable, and produces the colours, flavours and aromas that we associate and demand in fried, baked, roasted and toasted foods. However, food raw materials derived from grains, tubers, storage roots and beans have highly complex compositions, and it is not surprising that heating them to very high temperatures also produces some undesirable contaminants. Here, we have reviewed three contaminants, acrylamide, HMF and PAHs, that are of particular concern in wheat-derived products. In all three cases, food manufacturers can limit the formation of these contaminants through careful control of the manufacturing process and quality control of the final product. However, in the case of acrylamide and HMF, the efforts of manufacturers can be undone by the presence of unexpectedly high concentrations of precursors in the raw material. That might be difficult to address for HMF because reductions in fructose and glucose concentrations would have a broad impact on product flavour, colour and aroma. For acrylamide, however, reducing the concentration of the key precursor, free asparagine, should have less impact on product quality, and free asparagine is amenable to both genetic and agronomic approaches to reducing its concentration.

Acrylamide is a matter of great concern in the realm of food safety due to its classification as a probable carcinogen. With wheat being a staple crop in numerous regions around the world, it serves as a crucial component in many basic food products, and reducing the acrylamide-forming potential of wheat would reduce the exposure of millions of consumers to dietary acrylamide. The presence of acrylamide in food is also a major regulatory-compliance issue for food manufacturers based in or exporting to the European Union, with the prospect of Maximum Levels being imposed in the near future. Effectively tackling this issue is of utmost importance to protect public health and ensure that food businesses can continue to produce wheat-based products that comply with regulations and provide consumers with confidence and peace of mind while enjoying their daily staples.

In the quest to reduce acrylamide content in food products, crop management plays a critical role. By implementing best crop management practices, such as ensuring good disease control and adjusting nitrogen and sulphur fertiliser applications, spikes in free asparagine concentration can be avoided. Furthermore, advancements in biotechnology offer promising solutions for enhancing wheat varieties. The development of low asparagine, genome-edited lines, in particular, shows that step reductions in free asparagine concentration in wheat grain can be achieved. Employing advanced technologies and refining processing conditions can result in significant reductions in acrylamide formation during cooking and manufacturing processes, but any changes to processing methods will be more effective and give more consistent and predictable results with low asparagine raw material. A low asparagine starting point would also mean that manufacturers could reduce the levels of acrylamide in their products without compromising on taste and quality. It is essential, therefore, that the regulatory environment on genome editing and other biotechnological applications gives breeders confidence that they can start using new breeding technologies.

In conclusion, the combination of crop management practices, genomic and biotechnological advancements, and improved processing techniques offer a comprehensive approach to tackling the issue of acrylamide and other processing contaminants in wheat-based products. By proactively addressing this issue, the food industry and its supply chain can enhance food quality and safety, benefiting both producers and consumers alike. The ripple effects of these efforts extend beyond individual health to encompass broader positive impacts on food quality and overall human well-being. A concerted and proactive approach to reducing contaminant levels is an essential step towards building a safer and healthier food landscape for the future.

## Figures and Tables

**Table 1:** Indicative Values and Benchmark Levels (μg kg^−1^; parts per billion (ppb)) for acrylamide in food, set by the European Commission, and the possible new Benchmark and Maximum Levels that are under discussion.

Food	Indicative Value 2011	Indicative Value 2013	Benchmark Level 2017	Under Discussion	
				Benchmark Level	Maximum Level
Soft bread	150	80	50	50	75
Soft bread (other)		150	100		
Breakfast cereals: bran products, whole grain cereals, gun puffed grain	400	400	300	300	500
Breakfast cereals: wheat and rye-based		300	300	300	400
Breakfast cereals: maize, oat, spelt, barley and rice-based		200	150		
Biscuits	500	500	350	300	500
Crackers	500	500	400		
Crispbread	500	450	350	300	400
Gingerbread	-	1000	800		
Cereal-based baby foods	100	50	40		
Biscuits and rusks for infants and young children	250	200	150		

## Data Availability

No new data were created or analyzed in this study. Data sharing is not applicable to this article.
